# Cell therapies for viral diseases: a new frontier

**DOI:** 10.1007/s00281-024-01031-8

**Published:** 2025-01-02

**Authors:** David Nardo, Emileigh G. Maddox, James L. Riley

**Affiliations:** Department of Microbiology and Center for Cellular Immunotherapies, University of Pennsylvania, Philadelphia, PA 19104 USA

**Keywords:** Virus specific T cells (VST), Mesenchymal stem cells (MSC), CAR T cells

## Abstract

Despite advances in medicine and antimicrobial research, viral infections continue to pose a major threat to human health. While major strides have been made in generating vaccines and small molecules to combat emerging pathogens, new modalities of treatment are warranted in diseases where there is a lack of treatment options, or where treatment cannot fully eradicate pathogens, as in HIV infection. Cellular therapies, some of which are FDA approved for treating cancer, take advantage of our developing understanding of the immune system, and harness this knowledge to enhance, or direct, immune responses toward infectious agents. As with cancer, viruses that evade immunity, do so by avoiding immune recognition or by redirecting the cellular responses that would eradicate them. As such, infusing virus specific immune cells has the potential to improve patient outcomes and should be investigated as a potential tool in the arsenal to fight infection. The present manuscript summarizes key findings made using cellular therapies for the treatment of viral infections, focusing on the potential that these strategies might have in controlling disease.

## Introduction

The immune system evolved to effectively control infectious agent by limiting their replication, minimizing pathogenesis, and establishing memory to prevent future infections. However, some pathogens developed ways to escape immune responses and/or exploit immune dysfunction to inflict substantial morbidity and mortality. The impact of vaccines and small molecules on infectious diseases cannot be overstated, and these agents save countless lives daily. However, these are not sufficient to cure all individuals with infectious diseases, and thus new modalities must be considered to reduce the impact of infectious agents on human health.

In the past few decades, cellular therapy has emerged as the fourth pillar of cancer therapy, in addition to surgery, radiotherapy and chemotherapy [1]. In its most basic form, cellular therapy is the manufacturing and delivery of immune cells toward a desired immune outcome. Bone marrow transplant is generally considered the first cell therapy as these cells reconstitute an immune system after lethal irradiation or chemical ablation, but the ability of engineered T cells to cure B cell derived cancers has spurred much interest in using similar approaches to cure solid tumors, autoimmunity, and infectious diseases [2, 3]. Currently, all FDA approved T cell therapies redirect T cells to a disease target by transducing them with a vector that expresses either a chimeric antigen receptor (CAR) or a TCR that recognizes a particular peptide MHC complex [4–6]. While transgenic TCRs employ natural or near natural molecules to redirect T cells to a particular peptide-MHC target, such as HLA-A2 restricted epitope of NY-ESO-1 protein for the treatment of synovial sarcoma, a CAR is a synthetic construct that pieces together domains from immune proteins to create a unique immunoreceptor [7].

**Fig. 1:**
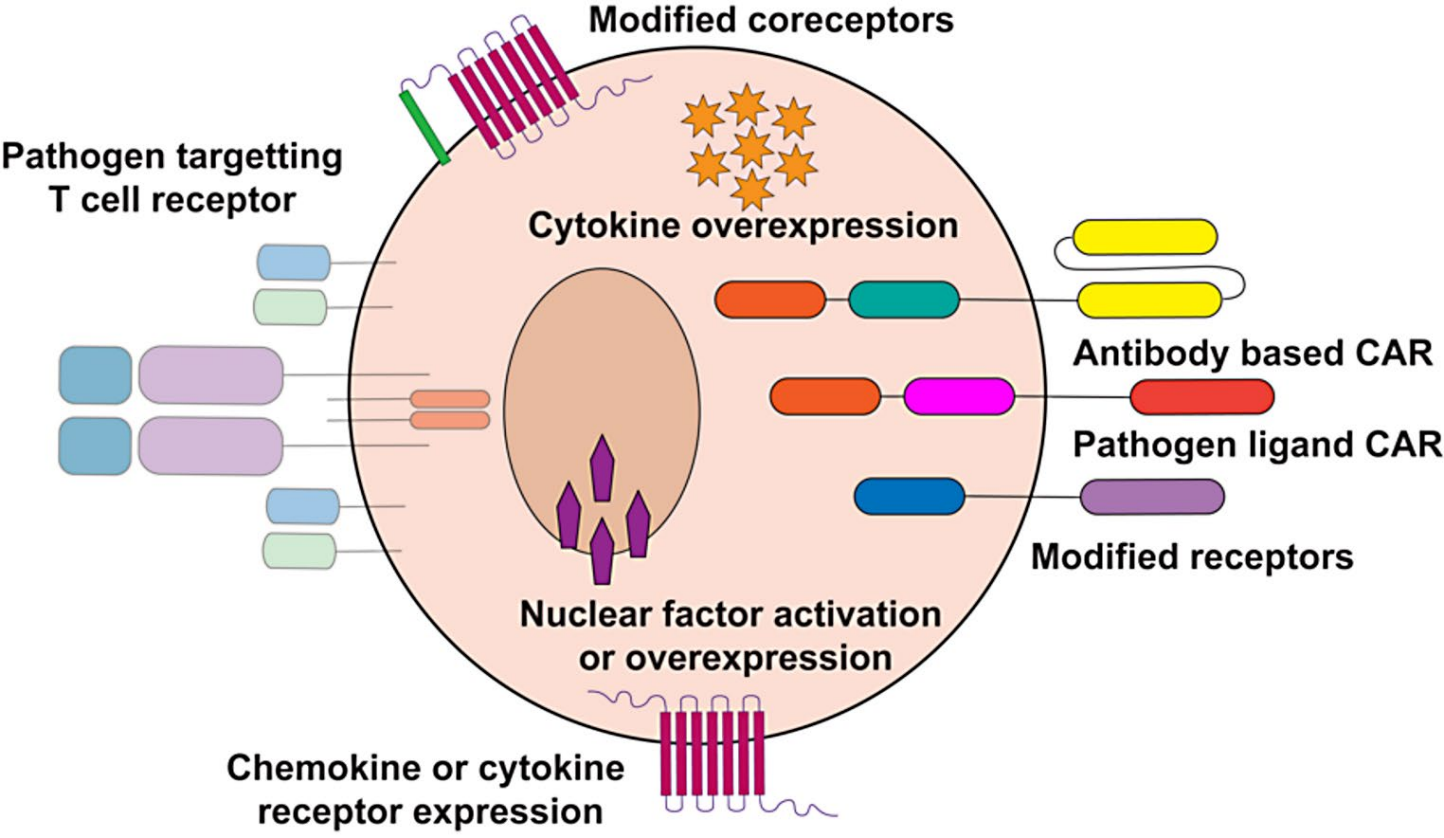
Genetic modification of pathogen targeting cell therapies. Cellular therapies can exploit the mechanisms that immune cells use to recognize pathogens through genetic modification. Some of these include the use of existing T cell receptors and chimeric antigen receptors (CARs) that recognize pathogens. Additionally, upregulation of various accessory proteins like chemokine or cytokine receptors, as well as modified co-receptors that improve cell targeting, effector functions and cell persistence have been investigated to improve cell therapy. Other additions include the over-expression of cytokines and nuclear factors that modify immune cell function

While many iterations of CAR T cells are being explored, the basic structure of a CAR links together a single chain variable fragment (scFV) targeting either a tumor, or pathogen, with at least one costimulatory domain (e.g.: CD28, 4-1BB, etc.), followed by the intracellular z domain of CD3 (CD3z) (Fig. 1) [2, 3, 6]. This composite protein redirects T cells to the new target and endows the cell with effector functions upon antigen recognition, without need for MHC presentation or co-receptor binding [1, 3–5]. The rationale behind using engineered immune cells for viral infections is similar, if not stronger, to that of using engineered cells for cancer therapy [1, 3, 8]. Viruses have evolved tactics to evade the immune system [9]. Moreover, since viruses present unique targets to engineered T cells, the risk of on target, off tissue toxicity is greatly reduced compared with tumor cells [10]. Engineering goes beyond redirecting cells to a particular target. Rather, genome editing approaches allow for the disruption of undesired genes and modification of genes to promote immunity. For instances, if a viral infection like RSV creates a milieu that promotes a TH2 response and this exacerbates lung damage, T cells could be engineered to resist TH2 differentiation and accentuate TH1 differentiation [11]. Moreover, additional cargo such as cytokines, chemokines, and cell surface markers can be engineered to be co-expressed with the CAR which can augment the persistence, trafficking, and function of the CAR T cells [2, 12, 13]. Ongoing efforts to safely expand and engineer immune cells in an affordable manner may make cellular therapy very attractive for treatment of infectious disease. In this review, we address three types of cellular therapy to fight viral infections: virus specific T cells, CAR T cells, and mesenchymal stem cells. We discuss how these are being studied to improve outcomes in viral diseases, where this has taken place, as well as potential areas where these might help to improve outcomes in patients.

## Viral infections

As highlighted by the SARS-CoV-2 pandemic of 2020, viruses pose a major threat to human health. Viruses vary greatly in structure, biology, and pathogenesis, with viral diseases ranging from mild infections that elicit little to no symptoms to more severe illness, like the lethal hemorrhagic fever associated with the Marburg and Ebola flaviviruses [14, 15]. Certain viruses, while not devastating in the short term, can establish chronic infections that deteriorate patient’s health over time. These include human immunodeficiency virus (HIV), hepatitis C virus (HCV), hepatitis B virus, herpes simplex viruses, Epstein Barr virus, coronaviruses (i.e.: long COVID), and others [16–18]. Novel viral infections can also emerge through exposure to new zoonotic carriers, or through mutations that generate novel variants, making viruses one of the most challenging pathogens affecting human health [19, 20].

### Virus specific therapies

To date, the most clinically successful cell therapy for infectious disease is virus specific T cell therapy (VST). Here, autologous virus-specific T cells are isolated and infused to prevent infections that affect bone marrow transplant recipients, like adenovirus, BK virus, cytomegalovirus (CMV), Epstein Barr virus (EBV), and JC virus. Because these patients undergo such extreme treatment modalities that leave them severely immunocompromised, the risks and cost associated with antiviral cell therapy are easily justified. As such, multiple trials have been conducted to assess outcomes of VST in HSCT patients [21].

**Fig. 2:**
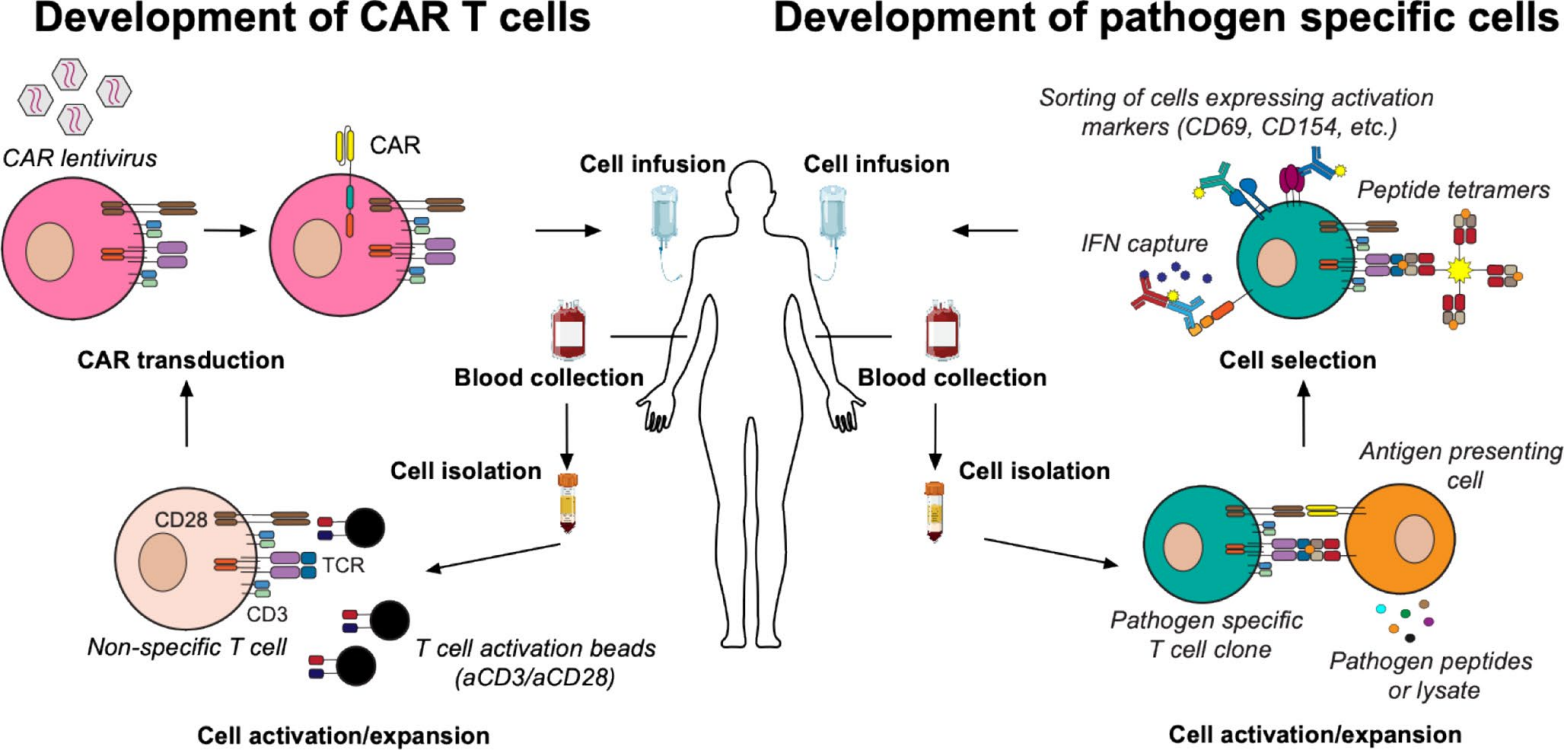
Development of cellular immunotherapies. In CAR T therapy (left), T cells isolated from blood are activated through engagement of CD3 and CD28, followed by CAR transduction, usually with lentiviral vectors, to produce chimeric receptors that induce T cell activation through direct contact with infected cells. These cells are then expanded and re-introduced into patients. Adoptive transfer of virus specific therapies (right) requires incubation of T cells with antigen presenting cells in the presence of viral peptides to allow expansion of reactive T cells that can be isolated based on activation markers, peptide tetramers, or interferon capture, before being infused

The first attempt to administer CMV responsive donor T cells after HSCT occurred in 1992, with transfer of lymphocytes expanded in the presence of CMV infected fibroblasts [22, 23]. These studies focused on recipient matched, CMV seropositive cells, and showed that this strategy was safe, did not cause graft versus host disease (GvHD), and allowed for reconstitution of CMV responses [22]. Later studies using CMV lysates pulsed with blood derived cells led to improvement in CMV specific T cells responses with varying degrees of clinical success. Since then, increasing understanding of T cell biology has led to modifications in VST manufacturing, such as shorter ex vivo culture periods, use of cytokine combinations to enhance cell responses, and optimization of peptides to expand highly cytolytic clones (Fig. 2) [21, 22, 24]. The process of clone isolation in VST manufacturing has also been optimized through several methods, including IFNg capture, where T cells are tagged with antibodies that also bind to IFNg in order to isolate peptide reactive cells, or sorting of antigen specific cells with peptide-HLA tetramers or activation markers of the T cell surface (Fig. 2) [24]. This is often followed by removal of alloreactive cells using these same techniques after coculture of donor cells with recipient APCs as well as new innovative strategies, discussed elsewhere, to increase the safety of the end product and prevent cell derived responses against recipients [21, 24–26].

A focus of recent studies has been multi-virus therapies aimed at common infections of HSCT patients [26]. In a recent trial by Papadoloulou et al. targeting adenovirus, EBV, CMV, BKV and HHV6 after HSCT, 8 of 11 recipients with active infection displayed no safety concerns related to VST and had an overall 94% response to infection [27]. While promising, the variability of this study makes it difficult to assess the effects of this therapy for each individual infection. However, clinical trials evaluating combination VST show that this strategy can induce immune responses against most or all pathogens tested and can expedite recovery from the viral infections in ~ 90% of cases [22]. In instances of donors who are virus naive, ‘off-the-shelf’ treatments from third party donors are also being explored. This strategy, validated by Haque et al. with partially matched EBV-CTLs, led to a 52% response at 6 months, with no adverse reactions [22, 28, 29]. Most recently, an open label phase II trial of AlloVir’s Posoleucel, an allogeneic, ‘off-the-shelf’ VST targeting adenovirus, BKV, CMV, EBV, HHV-6 and JCV in HSCT, showed this strategy was well tolerated with no therapy related toxicities, although three patients developed GvHD. After six weeks, 95% of participants responded to treatment, with a plasma viral load reduction of 97%. Unfortunately, the study was not directly compared against standard of care, an issue with many VST trials [30, 31].

In addition to HSCT related infections, VST has also been evaluated to treat other viral diseases. Zika virus, which is associated with severe birth defects in infants, has been targeted by Hanajiri, et al. whose work with polyclonal VST suggests that this might be a viable option for clinical evaluation [32]. HPV, HMPV, and RSV therapies have also been evaluated in preclinical models and shown positive results [21, 32–34]. Additionally, HPyV1 and HPyV2, the polyomaviruses responsible for progressive multifocal leukoencephalopathy, have been studied as VST targets [35–37]. This year, Tevogen Bio announced the results from their TVGN 489 trial, which employs HLA matched CD8 T cells enriched and expanded against COVID. Early results suggest that no major toxicities are associated with this treatment, including infusion reactions, cytokine release syndrome, neurotoxicity, or GvHD [38]. Rapa Therapeutics also developed an ‘off-the-shelf’ allogeneic product, RAPA-501-ALLO, that showed some promise but was removed from a phase II study due to reduced efficacy [39].

While VSTs show promise in transplant related infections, and possibly acute infections, efforts to use these in chronic HIV infection have not achieved the same clinical success. Some of the difficulties in eradicating HIV are highlighted in the section below. However, a major obstacle for autologous HIV VSTs is the potential for integration of replication competent virus in CD4 T cells, which makes them susceptible to viral and CTL mediated lysis during manufacturing [40]. Allogeneic transfer of cells from HIV elite controllers has been attempted, but these studies suggest that HIV specific CTLs induce epitope escape and a rapid decline of transferred CTLs, with variable results in disease outcomes (viral loads, cell numbers, etc.) [41–46]. In a recent study, people with HIV were treated with Vorinostat to shock the HIV reservoir out of latency and then infused with autologous HIV-specific T cells. While it is was feasible to manufacture these T cells and no significant adverse events were reported, the HIV reservoir was not reduced [47]. Given HIV propensity to evade the natural immune response, these early studies may suggest that an engineered T cell endowed with improved recognition, persistence and efficacy is required to eradicate HIV from individuals [48–51].

### Chimeric antigen receptors

Despite their role in human health, there are few treatment options for most viral diseases outside HIV or Hepatitis C (HCV). In HCV, direct-acting antiviral agents (DAA) can promote cures in up to 98% of those infected [52]. In HIV, however, anti-retroviral therapy (ART) allows individuals to live long lives without major health effects, but it is unable to eradicate the virus completely. For this reason, there is a large effort to develop cell and gene therapies that permanently cure HIV. These strategies have been reviewed extensively by us and others and thus we will only briefly discuss this topic [2, 3, 8, 44, 45, 53–57].

HIV possesses three attributes that make it particularly difficult to eliminate: a high mutation rate, which allows it to rapidly evade immune pressures; depletion of CD4 cells which impairs all immune responses and especially those targeting HIV since the HIV-specific CD4 T cells are preferentially infected by HIV; and lastly, HIV rapidly generates a latent reservoir that can re-emerge upon ART discontinuation [9, 53, 58–65]. Initial clinical attempts to use CAR T cells with first generation CD4 based CARs did not provide lasting infection control, despite evidence that the cells could persist in patients [66]. A more recent clinical study that infused bNAb based CAR T cells showed a slight delay of viral rebound after an analytical treatment interruption compared to historical controls and some evidence of viral escape to the CAR T cell [67]. Currently, our group is assessing the safety and efficacy of second generation CD4 CAR T cells modified by zinc finger nucleases (NCT03617198) and a group from UC Davis is testing a gp120 targeting CAR construct [68]. Results from these trials will hopefully provide further evidence for the safety of CAR therapies for HIV, as well as, understanding of the potential of these therapies at eradicating, or controlling, HIV infection.

CAR therapy has not been largely pursued for viral infections outside of HIV. This is likely because many infections are too acutely severe to allow to CAR manufacturing, not severe enough to warrant such costly treatment options, or establish latent disease without life threatening consequences. However, cell therapies targeting chronic latent infections from EBV, HSV or HPV can provide a way to fully eradicate these viruses. These approaches can take advantage of viral entry molecules, as with HIV and the CD4 CAR, but also with broadly neutralizing antibody against conserved structures on the viral surface, such as gH/gL/pUL128-pUL130-pUL131 for CMV or gB for HSV [69, 70]. Moreover, while CAR therapies might seem counter intuitive as a treatment option for acute infections due to the risk associated with therapeutic delays, ‘off-the-shelf’ treatments might offer way to overcome this obstacle similar to what is being contemplated for VST treatments.

### CAR tregs and mesenchymal stem cells

While some infections inhibit immune function to evade eradication, others like, influenza or coronavirus, can induce an overzealous immune response that can be lethal [71, 72]. In these scenarios, modulation of the immune response to decrease inflammatory damage, while maintaining pathogen eradication, is preferred. Engineered regulatory T cells have shown promise in inhibiting such immune responses [73]. Tregs can limit the amount of IL-2 present and thus dampen immune responses to influenza [74]. Tregs expressing a CAR are especially attractive as they cells have been shown to traffic to target tissue and become activated [75]. Additionally, Tregs play an important role in tissue repair and thus may not only prevent damage but may also assist in repairing damage that has already occurred [76].

In addition, mesenchymal stem cells (MSCs) derived from various donor sources, like placenta, adipose tissue, bone marrow and blood, are an attractive cellular therapy restore balance to the immune system and enhance tissue repair [71, 72, 77, 78]. As an ‘off-the-shelf’ therapies, MCSs can be frozen and stored for use in HLA matched donors. Additionally, MSCs have low levels of HLA-I, making them ideal for emergency treatment [79]. When used to treat influenza, MSCs from various tissues have shown reduction of proinflammatory molecules, alveolar fluid accumulation, and lung damage [71, 72, 77, 78, 80–82]. Interest in MSCs grew during the COVID19 pandemic, as their administration was shown to improve patient outcomes by reducing inflammatory damage and hospitalization [71, 72, 80, 83, 84]. These, in addition to the allogeneic ‘off-the-shelf’ VST strategies above, have allowed for cell based therapies to become much more widespread for treating viral infections and enhancing the role of cell therapies in the context of acute infections, where minimizing the time to treatment is essential.

## Conclusions

Despite years of effort and progress, viral diseases continue to play a major role in human health. While antiviral and vaccine researchers are pushing therapeutic boundaries, in many instances the inability of the immune system to respond effectively to pathogens ultimately leads to poor disease outcomes. Cellular immunotherapy, a field that has made major strides in the past few decades, may offer an intriguing and effective solution to overcome some of this issue by allowing for better targeting and responses to viral infections. Thus far, most major advancements in cellular therapies have been made in cancer with T cell-based strategies. However, there is much that can be learned by studying cell therapies for viral diseases.

While infection targeting cell therapies make most sense in chronic, so far uncurable diseases, like HIV, they also offer a promising way to advance the understanding of how current treatments can better eradicate infection reservoirs, and how immune cells function at these sites. Beyond viral infections, cellular therapies also have a role in other infections, including fungal and bacterial diseases, where both surface structures, as well as conserved pathogen molecules have served to target T cells to infected tissues [85–88]. T cells have also been the focus of cell therapies in cancer due to their longevity and their role in coordinating immunity. However, it’s becoming more evident that other cell types can be harnessed to enhance immunity in disease [12, 89]. Additionally, antimicrobial responses can be exploited to benefit cancer research. For instance, studying the effect of the lymphatic microenvironment on CAR T cell function may shed light on how these might be derailed in metastatic tumors. The same is true of other pathogens residing in immune-privileged sites, like zika virus.

Some of the obstacles to cellular therapies in infection are the time constraint created by acute infections, and the cost associated with generating such therapies. Cellular immunotherapies might not be feasible where validated, cost-effective, antivirals exist. However, as the role of cell therapies becomes better understood, predictive modelling of these systems can help expedite the process by which new treatments are evaluated and optimized, reducing the cost associated with these therapies [90–93]. Another issue in the field is the lack of homogeneity and underpowering of clinical studies, which makes it difficult to draw robust conclusions from clinical trials. As such, increasing the availability of these studies, perhaps through as ‘off-the-shelf’ cell products or in vivo nanoparticle based CAR systems, may help to increase access and allow for cell immunotherapies to become more appealing in the context of infection [30, 31, 94].

## Data Availability

Data sharing not applicable to this article as no datasets were generated or analyzed during the current study.
